# *Petroselinum crispum* (Parsley) Leaf Extract Attenuates Ethylene Glycol–Induced Nephrolithiasis Through Antioxidant and Renoprotective Effects

**DOI:** 10.1155/bmri/1306129

**Published:** 2026-07-08

**Authors:** Abdullah H. Al-Gamli, Abdullah H. Maad, Loai Aljerf, Khaled A. Alhumaidha, Bashier Osman, Mohammed E. Shayoub, Muaaz Alajlani

**Affiliations:** ^1^ Department of Pharmaceutical Sciences, Faculty of Pharmacy, University of Science and Technology, Sana′a, Yemen, ust.edu.ye; ^2^ Department of Pharmaceutics, College of Pharmacy, University of Al-Ameed, Karbala, Iraq; ^3^ Key Laboratory of Organic Industries, Department of Chemistry, Faculty of Science, Damascus University, Damascus, Syria, damascusuniversity.edu.sy; ^4^ Department of Pharmacology, Faculty of Pharmacy, University of Sana′a, Sana′a, Yemen; ^5^ Department of Pharmacology, Faculty of Pharmacy, University of Khartoum, Khartoum, Sudan, uofk.edu; ^6^ Faculty of Pharmacy, Arab International University, Damascus, Syria, aiu.edu.sy; ^7^ Faculty of Pharmacy, Syrian Private University, Damascus, Syria, spu.edu.sy

**Keywords:** calcium oxalate, kidney stone, nephrolithiasis, oxidative stress, *Petroselinum crispum*, phytotherapy, renal protection

## Abstract

Nephrolithiasis affects approximately 12% of the global population and is increasingly diagnosed at younger ages, highlighting the need for safer and more effective therapeutic options. Although *Petroselinum crispum* (*P. crispum*) is traditionally recognized for its diuretic properties, its specific curative efficacy against established calcium oxalate stones and its impact on the underlying oxidative stress pathways remain insufficiently characterized. Additionally, there is a critical need to validate the dose‐dependent renoprotective mechanisms of the plant′s extract to bridge the gap between ethnomedicinal use and clinical application. This study evaluated the renoprotective and antiurolithiatic potential of *P. crispum* ethanolic leaf extract in a well‐established rat model of ethylene glycol–induced nephrolithiasis. Male albino rats received 1% ammonium chloride and 0.75% ethylene glycol for 28 days to induce lithiasis. From Day 15 onward, two groups were treated orally with *P. crispum* extract at 300 or 600 mg/kg. Biochemical analyses of urine, serum, and kidney tissue, along with histopathological assessments, were performed. Ethylene glycol administration caused marked lithogenic changes, including decreased urine volume, elevated urinary pH, increased calcium excretion, oxidative stress, and impaired renal function. Moreover, it restored antioxidant status by elevating glutathione and reducing malondialdehyde and nitric oxide levels. Histopathological analysis confirmed a protective effect, with decreased crystal deposition and preservation of renal tubular architecture. These findings suggest that *P. crispum* exerts multimechanistic protective effects—diuretic, antioxidant, and nephroprotective—against urolithiasis. Further studies are warranted to elucidate the extract′s molecular mechanisms and validate its clinical potential as a plant‐based therapeutic agent for kidney stone prevention and management.

## 1. Introduction

Nephrolithiasis—also referred to as urolithiasis—is a globally prevalent urological disorder characterized by the formation of calculi within the renal tract, primarily due to the crystallization of solutes such as calcium and oxalate in the urine [1]. It affects approximately 12% of the global population and presents a high recurrence risk, with rates reaching up to 50% within 5–10 years and 75% over two decades [2]. Calcium oxalate stones account for more than 80% of cases, making them the most common subtype [3]. Nephrolithiasis not only imposes a significant burden on healthcare systems—with costs estimated in billions annually—but also severely compromises quality of life due to the associated pain and high recurrence rates [3]. This substantial economic impact underscores the urgent need for more cost‐effective and accessible therapeutic strategies.

The pathogenesis of nephrolithiasis is multifactorial, involving urinary supersaturation with lithogenic constituents, nucleation, crystal growth, aggregation, and retention, often exacerbated by oxidative stress, inflammation, and renal tubular injury [4]. Emerging evidence further suggests that environmental toxicants and metabolic stressors often act synergistically to compromise renal integrity, necessitating the exploration of bioactive nutraceuticals that can modulate host metabolic health and gut microbial architecture [5]. In recent decades, the incidence of nephrolithiasis has increased sharply, with onset now commonly reported at younger ages. This epidemiological shift has been attributed to modern dietary patterns, reduced physical activity, and environmental exposures [6], underscoring changes in disease dynamics. Additionally, sex and socioeconomic disparities have been documented: Men are more prone to calcium‐based stones, whereas infection‐related stones are more common in women [7]. Interestingly, higher incidence rates have also been reported among affluent populations, likely due to dietary habits rich in animal protein, sodium, and oxalate [8]. Although several pharmacological and surgical interventions—such as thiazide diuretics, potassium citrate, and extracorporeal shock wave lithotripsy—are available, none have demonstrated universal efficacy in preventing stone recurrence. Moreover, invasive interventions are often required in refractory cases [9]. These limitations have catalyzed the search for alternative, safer, and more cost‐effective therapeutic strategies. In particular, interest has grown in plant‐based treatments supported by traditional medicine systems [10].

*Petroselinum crispum* (*P*. *crispum*) (parsley), a medicinal and culinary herb belonging to the Apiaceae family, has been traditionally used for renal and urinary tract ailments [11]. Its essential oils—particularly apiol and myristicin—have been reported to possess diuretic, antioxidant, and antimicrobial properties [12]. Additionally, pharmacognostic studies have revealed the presence of phytoconstituents such as flavonoids, triterpenoids, and tannins in *P. crispum*, compounds known to exert nephroprotective, anti‐inflammatory, and crystal‐inhibitory effects. Flavonoids, for instance, are potent antioxidants that can neutralize reactive oxygen species (ROS) implicated in renal tubular injury [13, 14]. Although numerous medicinal plants have been investigated for their antiurolithiatic properties, robust in vivo validation of *P. crispum* using standardized and reproducible nephrolithiasis models remains limited. Previous studies have reported preliminary protective effects; however, they lack comprehensive evaluation of biochemical, oxidative stress, and histopathological parameters. Moreover, the mechanistic basis underlying its potential renoprotective effects has not been sufficiently elucidated. Therefore, there is a clear need for systematic experimental studies to validate the plant′s efficacy and explore its multitargeted mechanisms of action in established models of calcium oxalate nephrolithiasis. Sayed and Abdel Moatamed [15] reported preliminary protective effects of parsley in urolithiasis, yet their study lacked mechanistic depth and histopathological confirmation. Complementing this, Khan et al. [16] emphasized the critical need for high‐quality, reproducible animal studies evaluating traditional medicinal plants in established models of calcium oxalate lithiasis.

In response to these research gaps, the present study is aimed at systematically assessing the renoprotective and antiurolithiatic efficacy of *P. crispum* ethanolic leaf extract in a validated rat model of ethylene glycol–induced nephrolithiasis. The investigation evaluates its effects on urinary and serum biochemical indices, oxidative stress markers in renal tissues, and histological alterations in kidney architecture. So, it addresses key knowledge gaps by utilizing a validated hyperoxaluria model, quantifying lithogenic and antioxidant biomarkers, and evaluating dose‐dependent therapeutic effects of *P. crispum* extract. Thereby, to our knowledge, this study represents one of the first comprehensive in vivo evaluations integrating biochemical, oxidative stress, and histopathological assessments to investigate the antiurolithiatic potential of *P. crispum*. These findings are anticipated to offer evidence‐based justification for the plant′s traditional use and may support its development as a phytotherapeutic agent for nephrolithiasis, particularly in low‐resource settings where conventional therapies are less accessible.

## 2. Material and Methods

The selection of *P. crispum* for this investigation is predicated on its rich profile of flavonoids and polyphenols, which are hypothesized to interfere with the lithogenic process—not merely through diuresis, but by modulating the biochemical environment of the renal tubules to prevent crystal nucleation and attachment. All selected doses and treatment duration were guided by previously validated experimental protocols to ensure reproducibility, induction of stable nephrolithiasis, and reliable assessment of therapeutic efficacy without inducing excessive systemic toxicity.

### 2.1. Plant Material Collection and Authentication

Fresh leaves of *P. crispum* were collected from the Medicinal and Aromatic Plant Institute, National Research Center (NRC), Khartoum, Sudan. Botanical identification was carried out by expert taxonomists, and a voucher specimen (Voucher No. PC/2023/012) was deposited at the herbarium of the NRC for future reference.

### 2.2. Chemicals and Reagents

Ethylene glycol (≥ 99.5%) and ammonium chloride (analytical grade) were obtained from Sigma‐Aldrich (St. Louis, Missouri, United States). Ethanol (70%), Folin–Ciocalteu reagent, and sodium carbonate were of analytical grade and procured from Merck (Darmstadt, Germany). Diagnostic kits for biochemical assays, including urea (Cat. No. UR100), creatinine (CR200), calcium (CA100), magnesium (MG100), reduced glutathione (GSH100), malondialdehyde (MDA100), and nitric oxide (NO100), were obtained from Biodiagnostic Co. (Cairo, Egypt).

### 2.3. Experimental Animals

Thirty healthy adult male Wistar albino rats (weighing 130–150 g) were procured from the Animal House Unit, University of Khartoum, Sudan. Animals were acclimatized for 1 week and maintained under standard laboratory conditions: temperature 30°C ± 2°C, 12 h light/dark cycle, and relative humidity 55*%* ± 5*%*. Rats were provided with commercial rodent chow (Amar Factory, Sudan) and water ad libitum.

#### 2.3.1. Ethical Considerations

The research was approved by the Ethics Committee of the Faculty of Pharmacy, University of Khartoum, Khartoum, Sudan (Ethical Code No. FOP/2020/Animal‐012). All experimental protocols were conducted in compliance with the ethical guidelines for the use of animals in research in line with ARRIVE (Animal Research: Reporting of In Vivo Experiments) guidelines and the regulations of the research ethics committee of this university. Additionally, all animal experiments were conducted in accordance with protocols approved by the United States National Institutes of Health (NIH). The authors have fully complied with ethical issues, such as plagiarism, data fabrication, and double publication.

All procedures involving anesthesia and euthanasia were performed in accordance with approved ethical standards to minimize animal suffering.

### 2.4. Preparation of *P*. *crispum* Leaf Extract

Leaves were thoroughly washed with distilled water, air‐dried at ambient temperature, and ground into fine powder using an electric grinder. A total of 500 g of powdered material was subjected to cold maceration in 70% ethanol (1:10 *w*/*v*) for 72 h with intermittent shaking. The extract was filtered using Whatman No. 1 filter paper and concentrated under reduced pressure using a rotary evaporator (Heidolph, Germany) at 60°C–70°C. The semisolid residue was air‐dried to a constant weight and stored in airtight amber glass containers at 4°C until use.

### 2.5. Phytochemical Screening

Qualitative phytochemical screening was performed using standard methods described by Evans [13]. The tests and indicative reactions were as follows:

• Alkaloids: Dragendorff′s reagent (orange–red precipitate)
• Triterpenoids: Liebermann–Burchard reaction (green–blue coloration)
• Saponins: foam test (persistent froth)
• Flavonoids: lead acetate test (yellow precipitate)
• Glycosides: Keller–Killiani test (brown ring at interface)
• Anthraquinones: Borntrager′s test (pink–red color in alkaline layer)
• Tannins: Braymer′s test (white precipitate with gelatin solution)

This chemistry‐focused analysis is aimed at identifying the diverse array of secondary metabolites present in the extract, which are crucial for understanding its pharmacological properties.

### 2.6. Determination of Total Phenolic Content (TPC)

The quantitative determination of TPC utilized a standard chemical colorimetric assay, providing insights into the extract′s antioxidant capacity, a property intrinsically linked to its chemical composition.

TPC was measured following the Folin–Ciocalteu colorimetric method [14]. Briefly, 0.5 mL of the extract was mixed with 0.5 mL of Folin–Ciocalteu reagent (diluted 1:10 with distilled water) and incubated for 5–8 min at 25°C. Then, 2.0 mL of 7.5% sodium carbonate was added, and the final volume was adjusted to 8 mL with distilled water. After 2 h of incubation at room temperature, absorbance was recorded at 725 nm using a UV–Vis spectrophotometer (Shimadzu UV‐1800, Japan), applying fundamental principles of physical sciences related to light absorption and molecular concentration. Gallic acid was used as the standard, and results were expressed as milligrams of gallic acid equivalents per gram extract.

### 2.7. Induction of Urolithiasis and Treatment Protocol

Experimental urolithiasis was induced as per the method described by Sayed and Abdel Moatamed [15]. Except for the normal control group, all rats received 0.75% (*v*/*v*) ethylene glycol and 1% (*w*/*v*) ammonium chloride in their drinking water for 28 days to promote calcium oxalate stone formation [17–22]. This dosing regimen is widely employed in experimental models to induce sustained hyperoxaluria and reproducible calcium oxalate crystal deposition, with ammonium chloride facilitating early urinary acidification and renal tubular injury during the initial phase, thereby accelerating lithogenesis [23]. The 28‐day duration allows for progressive crystal nucleation, growth, and renal tissue damage, closely mimicking the pathophysiological progression of human nephrolithiasis [24]. The delayed initiation of treatment was designed to allow establishment of lithiasis prior to intervention, thereby enabling evaluation of the curative (rather than prophylactic) potential of the tested agents. Treatment regimens, administered orally from Day 15 to Day 28, were as follows:

• Group I: Normal control (saline, 5 mL/kg)
• Group II: Urolithiatic control (no treatment),
• Group III: Cystone‐treated (750 mg/kg; Himalaya Drug Co., India), a standard polyherbal antiurolithiatic formulation, was included as a reference drug at a dose commonly used in experimental nephrolithiasis studies to validate the model and benchmark therapeutic efficacy [25, 26].
• Group IV: *P. crispum* extract (300 mg/kg), and
• Group V: *P. crispum* extract (600 mg/kg).

The selected doses of *P. crispum* extract (300 and 600 mg/kg) were based on previously reported similar pharmacological studies on *Entada abyssinica* [27] demonstrating safety and biological efficacy of parsley extracts within this range. The use of two dose levels enabled assessment of dose‐dependent therapeutic responses while remaining within nontoxic limits established in earlier in vivo investigations [28].

### 2.8. Urine Collection and Analysis

On Day 28, rats were placed individually in metabolic cages for 24‐h urine collection with unrestricted access to water. Urine volume was measured using a graduated cylinder. pH was measured using a calibrated digital pH meter (Mettler Toledo SevenCompact, Switzerland). Urinary calcium and magnesium were quantified using colorimetric diagnostic kits (Biodiagnostic, Egypt) based on the *o*‐cresolphthalein complexone and calmagite methods, respectively [29].

### 2.9. Blood Collection and Serum Biochemistry

Following urine collection, animals were anesthetized using an intraperitoneal injection of ketamine (80 mg/kg) and xylazine (10 mg/kg), and blood samples (~1.5 mL) were collected via retro‐orbital puncture using heparinized capillaries. Serum was separated by centrifugation at 3000 rpm for 10 min at 4°C and analyzed for urea and creatinine levels using commercial kits (Biodiagnostic, Egypt) [30].

### 2.10. Renal Tissue Collection and Oxidative Stress Biomarkers

Following blood collection, animals were humanely euthanized by cervical dislocation under deep anesthesia (ketamine 80 mg/kg and xylazine 10 mg/kg, intraperitoneally). Both kidneys were then excised postmortem, rinsed in cold 1x phosphate‐buffered saline (PBS), blotted dry, and weighed.

Kidney tissues were homogenized in 1x PBS (20% *w*/*v*) using an MPW‐120 homogenizer (Med Instruments, Warsaw, Poland). Homogenates were stored at −20°C overnight and then subjected to two freeze–thaw cycles. Supernatants were obtained by centrifugation at 5000x g for 5 min at 4°C using a Sigma 2K15 refrigerated centrifuge (Sigma Laborzentrifugen GmbH, Germany), a process governed by physical sciences principles to separate components based on density. Levels of GSH, MDA (lipid peroxidation marker), and NO were quantified using biodiagnostic assay kits [30] as per the manufacturer′s protocols. It should be noted that the assessed oxidative stress parameters (GSH, MDA, and NO) represent biochemical markers of redox status rather than enzymatic activities, and their units are expressed accordingly per gram of tissue.

### 2.11. Histopathological Examination

Formalin‐fixed kidneys were dehydrated, embedded in paraffin, and sectioned at 5‐*μ*m thickness. Tissue sections were stained with hematoxylin and eosin (H&E) and examined under a Leica DM2500 light microscope (Leica Microsystems, Germany) equipped with a DFC450 camera. Microscopic evaluation of renal architecture and crystal deposition was conducted by a pathologist blinded to group allocations [31]. Semiquantitative histopathological scoring was performed to assess the severity of renal damage based on tubular degeneration, crystal deposition, and interstitial inflammation. Tissue alterations were graded on a scale of 0–3 as follows: 0 = *normal histology*, 1 = *mild changes* (minimal tubular damage and sparse crystal deposition), 2 = *moderate changes* (evident tubular degeneration, moderate crystal deposition, and inflammatory infiltration), and 3 = *severe changes* (extensive tubular damage, abundant crystal deposition, and marked inflammation), as previously described in experimental nephrolithiasis studies [32–34].

### 2.12. Statistical Analysis

For all quantitative parameters, individual data points corresponding to each animal (*n* = 6 per group) are presented alongside group means to provide a clear representation of data variability and distribution. So that data were expressed as mean ± standard error of the mean (SEM), and individual data points representing each animal were plotted to enhance transparency and illustrate data distribution within groups. Statistical analysis was performed using GraphPad Prism Version 9.0 (GraphPad Software, California, United States). One‐way analysis of variance (ANOVA) followed by Tukey′s Honestly Significant Difference (HSD) post hoc test was employed to assess differences among groups. A *p* value < 0.05 was considered statistically significant.

## 3. Results

### 3.1. Phytochemical Profiling and TPC of *P*. *crispum* Leaf Extract

Qualitative phytochemical screening of the ethanolic leaf extract of *P. crispum* confirmed the presence of several major classes of secondary metabolites. As shown in Table 1, the extract tested positive for triterpenoids, saponins, flavonoids, tannins, and alkaloids, whereas glycosides and anthraquinones were not detected.

**Table 1:** Phytochemical constituents of *Petroselinum crispum* leaf extract.

Phytoconstituents	*P. crispum* leaf extract
Triterpenoids	+
Glycosides	−
Anthraquinones	−
Saponins	+
Flavonoids	+
Tannins	+
Alkaloids	+

*Note:* + (presence) and − (absence).

Quantitative determination of TPC revealed a concentration of 21.27 ± 1.1 mg GAE/g dry extract.

### 3.2. Urine Biochemical Parameters

**Figure 1:**
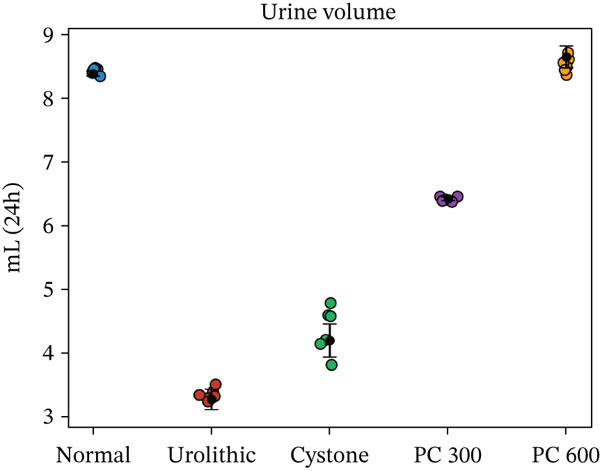
Scatter plot showing individual animal data points for 24‐h urine volume (mL/24 h) across experimental groups. Each dot represents one animal (*n* = 6 per group), with group mean ± SEM indicated.

The urine analysis is summarized in Table 2, and individual animal data for urine volume are presented in Figure 1. Ethylene glycol administration significantly reduced urine output, whereas treatment with *P. crispum* extract restored urine volume in a dose‐dependent manner (Figure 1). The urolithic control group showed significantly reduced 24‐h urine output (3.28 ± 0.16 mL; *p* < 0.05 vs. normal control) and elevated urinary pH (8.68 ± 0.05; *p* < 0.05). Administration of *P. crispum* extract at 300 and 600 mg/kg significantly increased urine volume (6.42 ± 0.03 mL and 8.64 ± 0.17 mL, respectively; *p* < 0.05 vs. urolithic control) and lowered pH values (8.23 ± 0.22 and 7.75 ± 0.08, respectively).

**Table 2:** Effects of *P. crispum* extract treatments on urine volume, pH, and levels of calcium and magnesium.

Parameters	Normal control	Urolithic control	Cystone (750 mg/kg)	*P. crispum* (300 mg/kg)	*P. crispum* (600 mg/kg)
Urine volume (mL/24 h)	08.38 ± 0.04	03.28 ± 0.16^a^	04.20 ± 0.26^a,b^	06.42 ± 0.03^a,b^	08.64 ± 0.17^b^
Urine pH	06.54 ± 0.08	08.68 ± 0.05^a^	06.38 ± 0.17^b^	08.23 ± 0.22^a^	07.75 ± 0.08^a,b^
Ca (mg/24 h)	34.59 ± 1.00	41.79 ± 0.84^a^	34.83 ± 0.84^b^	44.98 ± 1.77^a^	34.03 ± 0.33^b^
Mg (mg/24 h)	35.74 ± 0.46	30.78 ± 0.23^a^	34.59 ± 0.39^b^	30.14 ± 1.58^a^	36.50 ± 0.64^b^

*Note:* Data were expressed as mean ± SEM (*n* = 6), with individual animal data points presented in the corresponding graphical representations.

^a^Significantly different from normal control at *p* < 0.05.

^b^Significantly different from urolithic control at *p* < 0.05.

Urinary calcium levels were significantly increased in the urolithic control group (41.79 ± 0.84 mg/24 h; *p* < 0.05), whereas magnesium excretion was significantly reduced (30.78 ± 0.23 mg/24 h; *p* < 0.05). Treatment with *P. crispum* extract at 600 mg/kg significantly reduced urinary calcium (34.03 ± 0.33 mg/24 h) and increased magnesium levels (36.50 ± 0.64 mg/24 h; *p* < 0.05 vs. urolithic control).

The 28‐day ethylene glycol administration significantly reduced urine output compared with the normal control group, indicating impaired renal function.

### 3.3. Serum Biochemical Analysis

**Figure 2:**
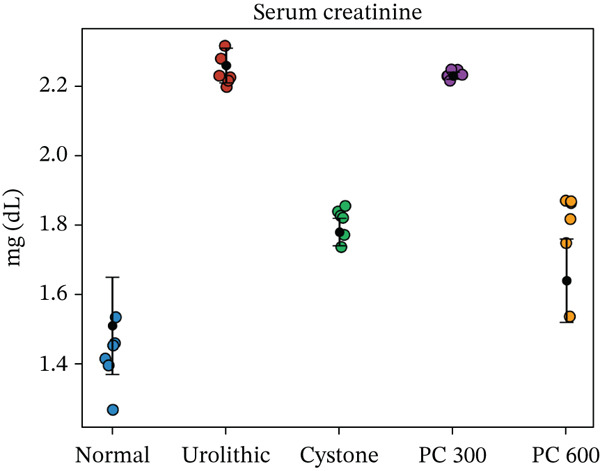
Scatter plot depicting individual serum creatinine levels (mg/dL) in experimental groups. Each point represents one animal (*n* = 6 per group), with mean ± SEM shown.

Serum creatinine and urea levels are presented in Table 3. In the urolithic control group, creatinine (2.26 ± 0.05 mg/dL) and urea (234.39 ± 2.90 mg/dL) levels were significantly elevated compared with the normal control group (*p* < 0.05), indicating impaired renal filtration function. Treatment with *P. crispum* extract reduced these levels in a dose‐dependent manner. At 600 mg/kg, creatinine and urea values were 1.64 ± 0.12 mg/dL and 96.82 ± 4.73 mg/dL, respectively (*p* < 0.05 vs. urolithic control). Individual variability in serum creatinine levels is illustrated in Figure 2.

**Table 3:** Effects of *P. crispum* extract treatments on serum creatinine and urea.

Parameters	Normal control	Urolithic control	Cystone (750 mg/kg)	*P. crispum* (300 mg/kg)	*P. crispum* (600 mg/kg)
Creatinine (mg/dL)	01.51 ± 0.14	02.26 ± 0.05^a^	01.78 ± 0.04^b^	02.23 ± 0.01^a^	01.64 ± 0.12^b^
Urea (mg/dL)	64.33 ± 1.99	234.39 ± 2.90^a^	68.47 ± 3.55^b^	142.04 ± 7.46^a,b^	96.82 ± 4.73^a,b^

*Note:* Data were expressed as mean ± SEM (*n* = 6), with individual animal data points presented in the corresponding graphical representations.

^a^Significantly different from normal control at *p* < 0.05.

^b^Significantly different from urolithic control at *p* < 0.05.

Consequently, ethylene glycol significantly increased creatinine levels, whereas *P. crispum* treatment restored renal function in a dose‐dependent manner.

### 3.4. Oxidative Stress Biomarkers in Renal Tissue

Levels of oxidative stress biomarkers reflecting MDA, NO, and nonenzymatic antioxidant status (GSH) in renal homogenates are summarized in Table 4. The urolithic control group showed a significant increase in MDA and NO levels (14.95 ± 0.39 nmol/g tissue and 105.19 ± 0.71 *μ* *m* *o* *l*/g tissue, respectively), and a significant decrease in reduced GSH; 2.35 ± 0.14 mg/g tissue compared with the normal control group (*p* < 0.05), indicating enhanced lipid peroxidation and oxidative stress.

**Table 4:** Effect of treatment with *P. crispum* leaf extracts (300 and 600 mg/kg) on renal GSH, MDA, and NO levels.

Parameters	Normal control	Urolithic control	Cystone (750 mg/kg)	*P. crispum* (300 mg/kg)	*P. crispum* (600 mg/kg)
GSH (mg/g tissue)	03.23 ± 0.06	02.35 ± 0.14^a^	02.54 ± 0.12^a^	02.52 ± 0.02^a^	02.92 ± 0.09^b^
MDA (nmol/g tissue)	06.83 ± 0.32	14.95 ± 0.39^a^	08.47 ± 0.49^a,b^	08.01 ± 0.10^b^	07.08 ± 0.24^b^
NO (*μ*mol/g tissue)	57.88 ± 2.48	105.19 ± 0.71^a^	85.48 ± 1.79^a,b^	96.44 ± 0.32^a,b^	66.06 ± 2.18^a,b^

*Note:* Data were expressed as mean ± SEM (*n* = 6), with individual animal data points presented in the corresponding graphical representations. Statistical analysis: one‐way ANOVA followed by Tukey′s post hoc test.

^a^Significant difference from normal control at *p* < 0.05.

^b^Significant difference from urolithic control at *p* < 0.05.

Treatment with *P. crispum* extract significantly modulated these markers. At 600 mg/kg, MDA and NO were reduced to 7.08 ± 0.24 nmol/g and 66.06 ± 2.18 *μ* *m* *o* *l*/g tissue, respectively, whereas GSH increased to 2.92 ± 0.09 mg/g tissue (*p* < 0.05 vs. urolithic control).

**Figure 3:**
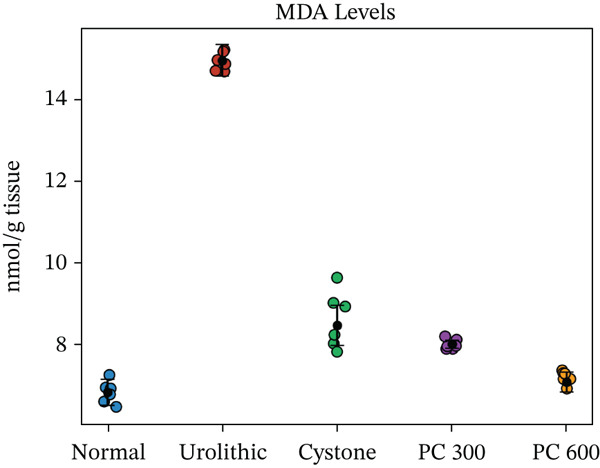
Scatter plot showing individual renal malondialdehyde (MDA) levels (nmol/g tissue) across experimental groups. Each dot represents one animal (*n* = 6 per group), with mean ± SEM indicated.

The distribution of individual MDA values is presented in Figure 3.

Ethylene glycol–induced a significant increase in lipid peroxidation, which was attenuated by *P. crispum* treatment in a dose‐dependent manner.

### 3.5. Histopathological Assessment of Renal Tissues

**Figure 4:**
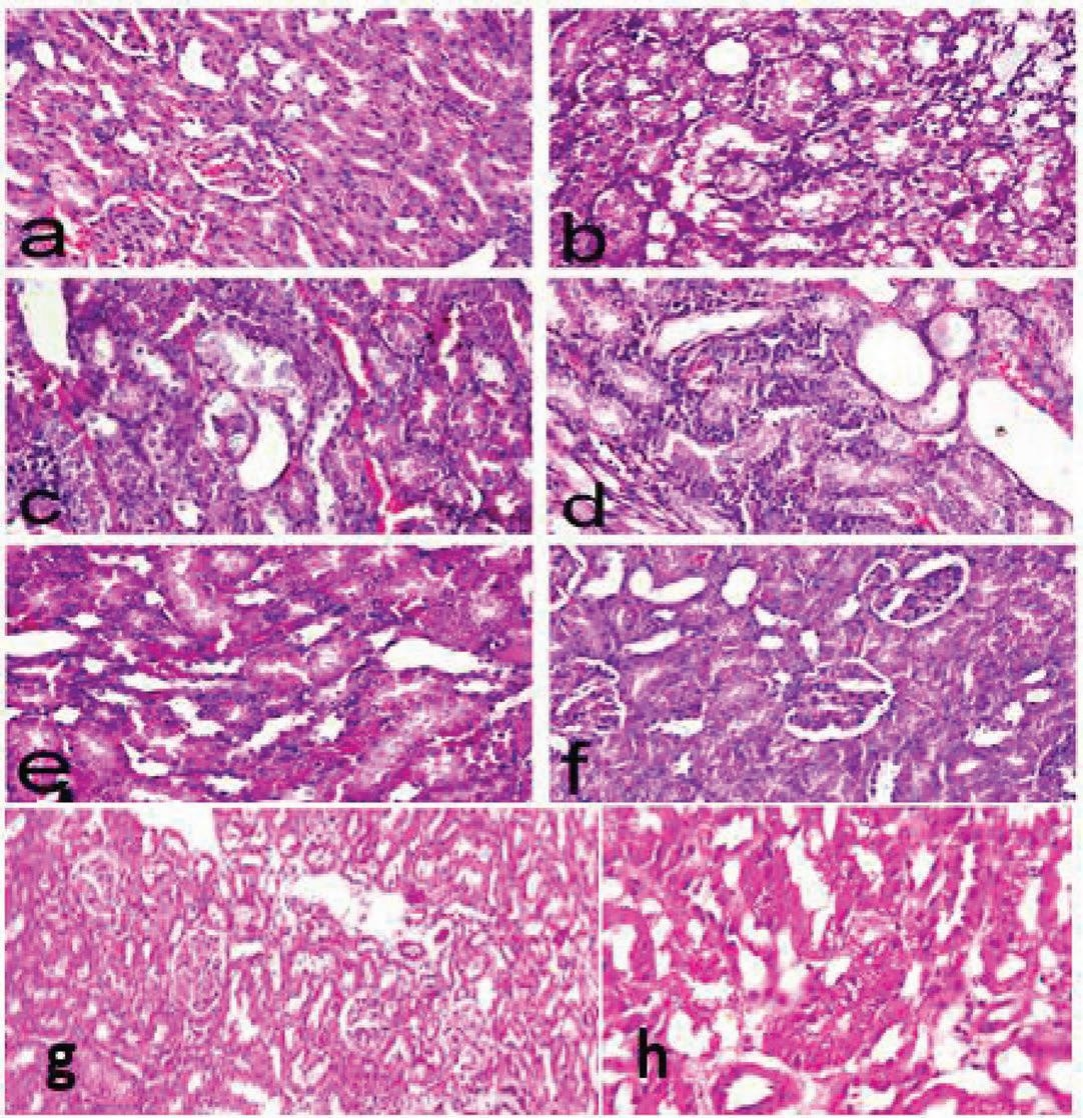
Representative photomicrographs of renal tissue sections stained with hematoxylin and eosin (H&E) (×400). (a) Normal control group showing intact glomeruli and normal tubular architecture; (b–e) urolithiatic control group showing (b) tubular epithelial degeneration, (c) calcium oxalate crystal deposition in tubular lumen, (d) interstitial inflammatory cell infiltration, and (e) tubular regeneration. (f) Cystone‐treated group showing mild tubular dilatation. (g) *P. crispum* (300 mg/kg)–treated group showing mild histological alterations with occasional crystal deposits. (h) *P. crispum* (600 mg/kg)–treated group showing near‐normal renal architecture with minimal degeneration and absence of crystal deposition.

Microscopic evaluation of H&E‐stained kidney sections revealed distinct histoarchitectural changes among the groups (Figure 4). Semiquantitative histopathological scoring demonstrated severe renal damage in the urolithiatic control group, whereas treatment with *P. crispum*, particularly at 600 mg/kg, markedly reduced the severity of tissue injury. Normal control rats exhibited normal renal glomeruli and intact tubular architecture. Urolithic control rats showed extensive vacuolar degeneration of the tubular epithelium, luminal calcium oxalate crystal deposits, tubular dilatation, and mononuclear cell infiltration in peritubular and interstitial regions. In rats treated with *P. crispum* extract at 300 mg/kg, mild tubular alterations and sporadic crystal deposits were observed. In contrast, kidneys from rats treated with 600‐mg/kg extract exhibited largely preserved renal morphology, with minimal vacuolar changes and absence of visible CaOx crystals.

## 4. Discussion

Nephrolithiasis is a multifactorial disorder resulting from an imbalance between promoters and inhibitors of crystal formation in the renal system. The ethylene glycol–induced hyperoxaluria model employed in this study effectively replicates calcium oxalate stone pathophysiology in humans and remains a gold standard for antiurolithiatic investigations [35, 36]. Male rats were used due to their increased susceptibility to stone formation, attributed to both lower urinary volume and hormonal factors—particularly androgen‐mediated suppression of osteopontin, a glycoprotein that inhibits crystal aggregation [16]. This sex‐based predisposition enhances the model′s sensitivity in evaluating therapeutic efficacy.

The current investigation comprehensively evaluated the renoprotective and antiurolithiatic efficacy of *P. crispum* leaf extract in this validated model. Phytochemical screening confirmed the presence of several bioactive classes, including flavonoids, triterpenoids, alkaloids, saponins, and tannins (Table 1). These constituents are known to possess diverse pharmacological activities relevant to nephrolithiasis modulation [37]. Flavonoids and tannins contribute to antiurolithiatic activity through calcium‐chelating and antioxidant properties, reducing urinary supersaturation and limiting oxidative damage to renal epithelial cells [16]. Meanwhile, alkaloids and triterpenoids are known to exert diuretic effects and inhibit the tubular reabsorption of lithogenic ions, potentially preventing crystal retention and facilitating their excretion.

Ethylene glycol administration resulted in hallmark features of nephrolithiasis, including reduced 24‐h urine output and elevated urinary pH (Table 2). These changes promote a lithogenic environment, which was counteracted by *P. crispum* through increased urine flow and enhanced crystal clearance. Treatment with *P. crispum*, especially at the 600‐mg/kg dose, significantly reversed these effects by enhancing diuresis and modulating pH toward physiological levels. These findings align with previous reports demonstrating the diuretic and natriuretic actions of *P. crispum*, contributing to reduced fluid retention and improved urinary clearance [38].

The inhibition of crystal nucleation and growth is heavily dependent on the balance of urinary ions. Disruptions in urinary calcium and magnesium levels were also observed in the urolithiatic control group, with hypercalciuria and hypomagnesiuria increasing the risk of crystal nucleation and aggregation. Magnesium is an established inhibitor of calcium oxalate crystallization, as it forms soluble complexes with oxalate and competes with calcium for binding sites [39]. The extract′s ability to reduce urinary calcium and restore magnesium excretion suggests that it may modulate renal ion transport pathways or interfere with crystal‐forming complexes, thereby restoring ionic equilibrium and minimizing lithogenic potential.

The rise in serum urea and creatinine in urolithiatic rats (Table 3) indicated compromised renal function, a common outcome of obstructive nephropathy. These elevations typically reflect tubular obstruction, glomerular filtration decline, and associated inflammatory responses. *P. crispum* administration significantly reduced both biomarkers in a dose‐dependent manner, indicating nephroprotection. This effect may be attributed to the antioxidant and anti‐inflammatory properties of the extract, which help preserve glomerular and tubular integrity under lithogenic stress.

Oxidative stress plays a central role in nephrolithiasis by promoting epithelial injury and crystal adhesion. It results from an imbalance between reactive oxygen and nitrogen species and antioxidant defenses. In this study, ethylene glycol increased MDA and NO levels while reducing GSH, indicating oxidative damage (Table 4). Treatment with *P. crispum* reversed these changes, demonstrating its antioxidant potential. Although specific pathways such as nuclear factor erythroid 2‐related factor 2 (Nrf2)/Kelch‐like ECH‐associated protein 1 (Keap1) [40, 41] were not directly assessed, the observed biochemical improvements suggest modulation of redox homeostasis. Furthermore, *P. crispum* treatment significantly mitigated these effects, reducing oxidative markers and restoring antioxidant capacity. The observed reduction in MDA and NO, coupled with increased GSH, highlights the extract′s potent antioxidant activity, a direct consequence of the chemical interactions of its phenolic compounds with ROS and nitrogen species, reflecting restoration of antioxidant balance [42]. These findings support the role of antioxidant activity in reducing crystal retention and renal injury [40, 41]. However, although enzymatic antioxidants such as SOD and GST were not directly quantified in the present study, the observed depletion of GSH and elevation of lipid peroxidation strongly suggest a compromised antioxidant defense system and enhanced free radical‐mediated cellular damage.

Histological examination of renal tissues corroborated the biochemical findings. The urolithiatic control group exhibited classic signs of oxalate‐induced injury, including vacuolar degeneration of tubular epithelium, crystal deposition, and interstitial inflammation (Figure 4). Rats treated with *P. crispum*, particularly at 600 mg/kg, displayed nearly preserved renal architecture with minimal vacuolation and an absence of visible CaOx deposits. These morphological improvements mirror the restoration of biochemical parameters and suggest that the extract not only prevents crystal formation but also mitigates tissue‐level damage. Comparable histopathological protection has been observed with other medicinal plant extracts possessing antiurolithiatic properties [18].

The antiurolithiatic activity of *P. crispum* appears to stem from a synergistic interplay of mechanisms:

• Diuretic effect → increased urine output and crystal elimination
• Ion modulation → reduced calcium, increased magnesium
• Antioxidant activity → reduced oxidative stress
• Tissue protection → preservation of renal architecture

These multitargeted effects highlight the therapeutic potential of *P. crispum* as a plant‐based agent for the prevention and management of nephrolithiasis. The findings also support its traditional use in renal disorders and suggest its relevance as a complementary strategy, particularly in regions where access to conventional therapies is limited.

Although the present study provides important preliminary evidence supporting the renoprotective and antiurolithiatic potential of *P. crispum*, several limitations should be acknowledged. First, the use of a rodent model—although well‐established for nephrolithiasis research—poses inherent constraints on translational relevance. Differences in renal physiology, xenobiotic metabolism, and hormonal regulation between rodents and humans may affect both the pharmacokinetics and pharmacodynamics of plant‐derived compounds. Thus, direct extrapolation of the results to human clinical settings should be approached with caution. Second, the investigation employed a crude ethanolic extract of the whole plant, without isolating, characterizing, or quantifying specific bioactive constituents. This lack of phytochemical resolution limits the mechanistic interpretation of the findings, as the individual contributions—or possible synergistic or antagonistic interactions—of the underlying compounds remain undefined. Third, the experimental duration was relatively short and did not encompass long‐term exposure, precluding assessment of chronic toxicity, metabolic degradation, and pharmacological interactions with standard urological therapies or other drugs. These aspects are critical for evaluating long‐term safety, therapeutic stability, and compatibility in a real‐world context. Fourth, although two extract doses were tested, a full dose–response analysis was not undertaken. This restricts the ability to define optimal therapeutic windows, establish minimum effective concentrations, or determine maximum tolerated doses—parameters that are vital for eventual clinical development. Fifth, although key oxidative stress markers such as GSH, MDA, and NO were assessed, additional enzymatic antioxidant parameters—including SOD, catalase (CAT), and GST—were not evaluated. The inclusion of these markers would have provided a more comprehensive understanding of the antioxidant defense system and deeper mechanistic insight into the renoprotective effects of *P. crispum*. Sixth, mechanistic studies at the molecular level are also imperative. Investigations into the regulation of renal transporters (e.g., Na^+^/K^+^‐ATPase and calcium channels), inflammatory markers (e.g., tumor necrosis factor‐alpha [TNF‐*α*] and interleukin‐1 beta [IL‐1*β*]), and oxidative stress signaling pathways (e.g., Nrf2/Keap1, and NF‐*κ*B) will provide valuable insight into how *P. crispum* modulates urolithogenesis. Such work will help delineate target‐specific actions and may uncover novel therapeutic pathways. Equally critical are comprehensive toxicological assessments covering acute, subchronic, and chronic exposures, as well as pharmacokinetic and bioavailability studies to inform appropriate dosing regimens. The application of in silico modeling and molecular docking techniques can further elucidate compound–target interactions and optimize candidate molecules for drug development. Seventh, to advance the clinical relevance of *P. crispum*, further investigation should adopt an integrated, multidisciplinary strategy. High‐resolution phytochemical profiling, including liquid chromatography–mass spectrometry (LC–MS) and nuclear magnetic resonance (NMR) spectroscopy, is essential to isolate and characterize the specific bioactive constituents. Bioactivity‐guided fractionation should accompany these efforts to prioritize fractions with the strongest antiurolithiatic activity. Addressing these areas will be essential for substantiating the therapeutic promise of *P. crispum* and advancing it toward safe and effective clinical application.

At last, the encouraging findings of this study, including significant improvements in urinary biochemical parameters, renal function markers, oxidative stress indices, and histopathological outcomes, substantiate the therapeutic promise of *P. crispum* as a natural intervention for calcium oxalate nephrolithiasis. This study provides a comprehensive in vivo evaluation combining lithogenic, oxidative, and histopathological assessments to demonstrate the dose‐dependent antiurolithiatic efficacy of *P. crispum*, thereby contributing novel experimental evidence to the field of phytotherapy in renal disorders. However, future studies should focus on isolating active compounds, elucidating molecular mechanisms, evaluating long‐term safety, and conducting clinical trials to confirm therapeutic efficacy.

## 5. Conclusion

This study provides compelling experimental evidence that *P*. *crispum* leaf extract exerts significant renoprotective and antiurolithiatic effects in a rat model of ethylene glycol–induced nephrolithiasis. Notably, treatment with the extract—particularly at 600 mg/kg—resulted in a marked increase in urine volume, normalization of urinary pH, and significant reduction in urinary calcium excretion, alongside restoration of magnesium levels. Furthermore, the extract significantly improved renal function markers, as evidenced by reductions in serum creatinine and urea levels, and attenuated oxidative stress by decreasing malondialdehyde and nitric oxide levels while restoring reduced glutathione content. Histopathological findings corroborated these biochemical improvements, demonstrating reduced crystal deposition and preservation of renal tubular architecture. Collectively, these findings highlight the multitargeted mechanism of action of *P. crispum*, involving diuretic, antioxidant, and nephroprotective effects, thereby addressing key pathogenic events in nephrolithiasis. The integration of biochemical, oxidative stress, and histopathological endpoints in this study provides a comprehensive in vivo validation of its antiurolithiatic potential, underscoring its novelty compared with previously limited reports. These results not only substantiate the traditional use of *P. crispum* but also position it as a promising candidate for the development of plant‐based therapeutic interventions. Future studies should focus on bioactive compound isolation, molecular pathway elucidation—particularly involving redox‐sensitive signaling—and well‐designed clinical trials to facilitate its translation into evidence‐based nephrolithiasis management.

## Author Contributions

A.H.A.‐G. performed formal analysis and contributed to methodology, funding acquisition, project administration, resources, and software. A.H.M. conceptualized the study, conducted data curation and investigation, and supervised the work, as well as contributed to resources, software, and manuscript review and editing. L.A. participated in the investigation, validation, and visualization, and contributed to drafting the manuscript and its subsequent revision. K.A.A. was involved in the investigation and contributed to resources, software, and manuscript review and editing. B.O. contributed to conceptualization and methodology. M.E.S. was responsible for data curation and visualization. M.A. contributed to validation and participated in manuscript drafting and review and editing.

## Funding

No funding was received for this manuscript.

## Conflicts of Interest

The authors declare no conflicts of interest.

## Data Availability

The data that support the findings of this study are available from the corresponding authors upon reasonable request.
